# Polyunsaturated Fatty Acid Metabolism Signature in Ischemia Differs from Reperfusion in Mouse Intestine

**DOI:** 10.1371/journal.pone.0075581

**Published:** 2013-09-20

**Authors:** Thomas Gobbetti, Pauline Le Faouder, Justine Bertrand, Marc Dubourdeau, Elisabetta Barocelli, Nicolas Cenac, Nathalie Vergnolle

**Affiliations:** 1 Inserm, U1043, Toulouse, France; 2 CNRS, U5282, Toulouse, France; 3 Université de Toulouse, UPS, Centre de Physiopathologie de Toulouse Purpan (CPTP), Toulouse, France; 4 William Harvey Research Institute, Barts and The London School of Medicine, Queen Mary University of London, Charterhouse Square, London, United Kingdom; 5 Lipidomic Core Facility, Metatoul Platform, Université Paul Sabatier, Toulouse, France; 6 Ambiotis-SAS, Canal Biotech 2, Toulouse, France; 7 Department of Pharmacy, University of Parma, Parma, Italy; McMaster University, Canada

## Abstract

Polyunsaturated fatty acid (PUFA) metabolites are bioactive autoacoids that play an important role in the pathogenesis of a vast number of pathologies, including gut diseases. The induction and the resolution of inflammation depend on PUFA metabolic pathways that are favored. Therefore, understanding the profile of n-6 (eicosanoids)/n-3 (docosanoids) PUFA-derived metabolites appear to be as important as gene or protein array approaches, to uncover the molecules potentially implicated in inflammatory diseases. Using high sensitivity liquid chromatography tandem mass spectrometry, we characterized the tissue profile of PUFA metabolites in an experimental model of murine intestinal ischemia reperfusion. We identified temporal and quantitative differences in PUFA metabolite production, which correlated with inflammatory damage. Analysis revealed that early ischemia induces both pro-inflammatory and anti-inflammatory eicosanoid production. Primarily, LOX- (5/15/12/8-HETE, LTB_4_, LxA_4_) and CYP- (5, 6-EET) metabolites were produced upon ischemia, but also PGE_3_, and PDx. This suggests that different lipids simultaneously play a role in the induction and counterbalance of ischemic inflammatory response from its onset. COX-derived metabolites were more present from 2 to 5 hours after reperfusion, fitting with the concomitant inflammatory peaks. All metabolites were decreased 48 hours post-reperfusion except for to the pro-resolving RvE precursor 18-HEPE and the PPAR−γαμμα agonist, 15d-PGJ_2_. Data obtained through the pharmacological blockade of transient receptor potential vanilloid-4, which can be activated by 5, 6-EET, revealed that the endogenous activation of this receptor modulates post-ischemic intestinal inflammation. Altogether, these results demonstrate that different lipid pathways are involved in intestinal ischemia-reperfusion processes. Some metabolites, which expression is severely changed upon intestinal ischemia-reperfusion could provide novel targets and may facilitate the development of new pharmacological treatments.

## Introduction

Polyunsaturated fatty acids (PUFAs) metabolites have been implicated in a vast number of inflammatory conditions, where they have potent bioactive signalling capacity [1]. Depending on the nature of the PUFA metabolites, and the timing of their release, they can either foster pro-inflammatory signals, or on the contrary, engage the inflammatory response towards a resolution phase and a return to homeostasis [2]. PUFA metabolites that are implicated in inflammation include eicosanoids derived from the n-6 arachidonic acid (AA) metabolic cascade (through the activation of the cyclooxygenase (COX), lipoxygenase (LOX) or cytochrome P450 pathways), but also eicosanoids derived from the n-3 eicosapentaenoic acid (EPA) metabolic cascade, and docosahexaenoic acid (DHA) metabolites. The consequences linked to an inflammatory event have been proven to be highly dependent on the metabolic pathways that are favoured [3].

While most of PUFA metabolites have been studied individually in an inflammatory context, it has been more difficult to investigate their presence and role as a whole in inflamed tissues, and to get a clear picture of the lipid metabolic cascades that are favoured in inflammation-related pathologies. The development of new techniques of liquid chromatography-tandem mass spectrometry (LC-MS/MS) now allows detecting simultaneously the presence of a number of PUFA metabolites in tissues, therefore leading to a better comprehension of disease-associated lipidic metaboloma. Here, we used this technique to run a wide analysis of the PUFA metabolites present in a model of intestinal ischemia-reperfusion in mice.

Intestinal ischemia-reperfusion injury (IRI) is a pathological event ensuing from a transient interruption of blood supply to the gut. This results in mucosal damage (known as ischemia-reperfusion injury) mediated by infiltration of neutrophils, platelet aggregation, vasodilation/vasoconstriction and the release of several inflammatory mediators [4], [5]. The presence and potential important role of PUFA metabolites in IRI is mainly supported by two observations. (1) Phospholipase A_2_, an enzyme that is responsible for the release of AA (one of the PUFA metabolite precursors) is strongly activated upon IRI [6], [7]. (2) Inhibitors of the COX and LOX metabolic pathways modify IRI-associated inflammatory damage [8]. Here, we have thus investigated the profile of PUFA metabolites that are released in the small intestine during tissue ischemia, and then upon reperfusion of intestinal artery, identifying the associated inflammatory damage. We have observed that the temporal progress of intestinal IRI is associated with different lipid metabolic patterns.

## Materials and Methods

The experimental protocol was approved by the Midi-Pyrenees Animal Care and Ethic Committee and was registered under the number MP/06/12/02/12 to the National Committee of Ethics and Animal Experimentation. It followed the guidelines of French Councils on Animal Care.

### Animals

C57Bl6 male mice (6–8 weeks-old) were obtained from Janvier (Le Genest Saint Isle, France). Animals were kept under pathogen-free conditions and were given free access to food and water.

### Surgical Procedures

Mice were anaesthetized with sodium pentobarbital (50 mg/kg i.p.). Following abdominal laparotomy, the small bowel was retracted to the left and the superior mesenteric artery was temporarily occluded using a microvascular clip to cause ischemia. After 50 minutes the clip was gently removed allowing reperfusion. The abdominal wall was closed by two-layer sutures. Following surgical procedure, mice were sacrificed by cervical dislocation right after the ischemic period (time 0), or 2, 5, 24 and 48-hours after reperfusion. Sham-operated (SO) animals, in which abdominal laparotomy and artery isolation were performed without occlusion of the vessel, served as controls for each reperfusion time point. For biochemical analysis jejunal tissues were excised and stored in liquid nitrogen before being processed. To investigate the role of transient receptor potential vanilloid-4 (TRPV4) endogenous stimulation during intestinal ischemia reperfusion a set of experiments (50-min. ischemia followed by 5 hours reperfusion) was performed, administering the selective (TRPV4) antagonist HC-067047 (50 mg/kg i.p. in 1% DMSO/1% Tween80/saline) 10-min before ischemia.

### Survival Rates

The survival rates in each group were monitored from the beginning of the surgery to the end of reperfusion times.

### Myeloperoxidase Activity (MPO)

MPO activity was measured as an index of granulocyte infiltration as previously described in jejunal tissues harvested at the time of sacrifice [9]. Briefly, jejunal tissue samples were homogenized in a solution of 0.5% hexadecyltrimethylammonium bromide dissolved in phosphate buffer solution (pH = 6) using Precellys®24 homogeniser in Precellys lysing CK14 tubes (Bertin Technologies). The homogenized tissues were centrifuged at 13,000×*g* for 5 minutes (at 4°C) and the supernatants were placed on 96 well plates. Buffer, supplemented with 1% hydrogen peroxide/O-dianisidine dihydrocholoride, was added to each well. Optical density readings were taken for 3 minutes at 30 seconds intervals at 450 nm using a microplate reader NOVOstar™ (BMG Labtech). Activity was normalized to the sample protein concentration determined with a BCA kit® (Pierce) and expressed as mU/mg protein.

### Assessment of Tissue Damage: Microscopic Damage Score

Specimens of the ileum were collected from the different groups of animals at the end of the perfusion period, in order to determine the level of tissue damage. Following overnight fixation in 10% formalin, specimens of the ileum were embedded in paraffin. Sections (5 µm) were stained with hematoxylin and eosin. Microscopic histological damage score was evaluated by a person unaware of the treatments and was based on a semiquantitative scoring system in which the following features were graded: extent of destruction of normal mucosal architecture (0, normal; 1, 2, and 3, mild, moderate, and extensive damage, respectively), presence and degree of cellular infiltration (0, normal; 1, 2, and 3, mild, moderate, and transmural infiltration), extent of muscle thickening (0, normal; 1, 2, and 3, mild, moderate, and extensive thickening), presence or absence of crypt abscesses (0, absent; 1, present), and presence or absence of goblet cell depletion (0, absent; 1, present). The scores for each feature were then summed with a maximum possible score of 11 as previously described [9], [10].

### Eicosanoid Extraction from Jejunal Tissue

Tissues were stored in liquid nitrogen until extraction. The extraction protocol is a modification of Le Faouder et al. ( [11]). For extraction, each frozen jejunal tissue sample was crushed with a FastPrep®-24 Instrument (MP biomedical) in 500 µL of HBSS (Invitrogen) and 15 µL of internal standard mixture (Deuterium-labeled compounds) (400 ng/mL). After 2 crush cycles (6.5 m/s, 30 s), 10 µL were withdrawn for protein quantification and 1 mL of cold methanol (MeOH) was added. Samples were centrifuged at 900 g for 15 min at 4°C. Supernatants were collected, diluted in HCl 0.02 M (10 mL) and submitted to solid-phase extraction on C18 cartridge 200 mg, 15 mL (Macherey Nagel). Briefly, columns were conditioned by successive passage of MeOH (10 mL) and HCl 0.02 M -MeOH 10% (10 mL). Each sample was loaded at a flow rate of about 1 drop per 2 s. After complete loading, columns were washed with HCl 0.02 M/MeOH-10% (5 mL). After drying under aspiration, lipid mediators were eluted with methyl formate (5 mL). After solvent evaporation under nitrogen gas, samples were dissolved with MeOH and stored at −80°C for Liquid chromatography/tandem mass spectrometry measurements.

### Liquid Chromatography/tandem Mass Spectrometry Measurements

By this technique we performed the quantification of 6-keto-prostaglandin F1α (6kPGF_1α_), thromboxan B2 (TXB_2_), prostaglandin E2 (PGE_2_), prostaglandin E3 (PGE_3_), prostaglandin A1 (PGA_1_), 8-iso prostaglandin A2 (8-isoPGA_2_), 15-Deoxy-Delta12,14-Prostaglandin J2 (15d-PGJ_2_)_,_ lipoxin A4 (LxA_4_), resolvin D1 (RvD_1_), leukotrien B4 (LTB_4_), leukotrien B5 (LTB_5_), 10(S), 17(S)-protectin (PDx), 18-hydroxyeicosapentaenoic acid (18-HEPE), 15-hydroxyeicosatetraenoic acid (15-HETE) and 12-HETE, 8-HETE, 5-HETE, 17-hydroxy-docosahexaenoic acid (17-HDoHE) and 14-HDoHE, 14,15-epoxyeicosatrienoic acid (14,15-EET) and 11,12-EET, 8,9-EET, 5,6- EET, 5-oxoeicosatetraenoic acid (5-oxo-ETE) in mouse intestinal tissue. To simultaneously separate 24 lipids of interest and 3 deuterated internal standards (LxA_4_-d5, LTB_4_-d4, 5-HETE-d8), LC-MS/MS analysis was performed on HPLC system (Agilent LC1290 Infinity) coupled to Agilent 6460 triple quadrupole MS (Agilent Technologies) equipped with electro-spray ionization operating in negative mode. Reverse-phase HPLC was performed using ZorBAX SB-C18 column (2.1 mm;50 mm;1.8 µm) (Agilent Technologies) with a gradient elution. Mobile phase A consisted of water, ACN and FA (75/25/0.1); Solvent B: ACN, FA (100/0.1). Compounds were separated with a linear gradient to 85% B from 0 to 8.5 min and 100% B to 9 min. Isocratic elution continued for 1 min at 100% B then 100% A was reached at 11 min and maintained to 12 min. The flow rate was 0.35 mL/min. The autosampler was set at 5°C and the injection volume was 5 µL.

### Standards

Standards: all compounds were mixed together in MeOH to a first calibration solution of 2000 ng/mL. Then, a series of dilution were prepared in MeOH (1000 ng/mL, 500 ng/mL, 250 ng/mL, 125 ng/mL, 62.5 ng/mL, 31.25 ng/mL, 15.6 ng/mL, 7.8 ng/mL, 3.9 ng/mL). IS was added to each level at a final concentration of 200 ng/mL. This lead to 10 working calibration standards at 500 ng/mL, 250 ng/mL, 125 ng/mL, 62.5 ng/mL, 31.25 ng/mL, 15.6 ng/mL, 7.8 ng/mL, 3.9 ng/mL, 1.95 ng/mL and 0 ng/mL containing 200 ng/mL of IS.

Data were acquired in MRM mode with optimized conditions (fragmentors and collision energy). Peak detection, integration and quantitative analysis were done using Mass Hunter Quantitative analysis software (Agilent Technologies).

### Cytokines Protein Expression

Jejunal tissue samples harvested at the time of sacrifice were homogenized in 700 µL of cell lysis buffer (20 mM Tris-HCl, pH = 7.5, 150 mM NaCl, 1 mM Na_2_EDTA, 1 mM EGTA, 1% Triton X-100, 2.5 mM sodium pyrophospate, 1 mM beta-glycerophospate, 1 mM Na_3_VO_4_, 1 g/mL leupeptin; Sigma) supplemented with anti-proteases cocktail (Sigma-fast) using Precellys®24 homogeniser in Precellys lysing CK14 tubes (Bertin Technologies). After centrifugation (10,000×*g* 10 min, 4°C), supernatants were filtered on QIAshredder columns (Qiagen, France). Fifty micro litres of this homogenate were used for simultaneous dosage of KC (keratinocyte chemoattractant), MCP-1 (Monocyte chemoattractant protein), and IL-6 (Interleukin-6) using cytometric bead array (CBA) on fluorescent cell sorter FACSCalibur, according to the manufacturer’s instructions (BD Biosciences, Le Pont de Claix, France) Raw values were normalized to the sample protein concentration determined with a BCA 8 kit® (Pierce). Cytokines concentrations were extrapolated from standard curves with the help of FCAP Array® software and expressed as pg/mg protein. In accordance with the manufacturer’s information, only values above the limit of cytokine detection were considered.

### Statistical Analysis

Data were analysed by the Student’s t-test for paired data or one-way ANOVA followed by Dunns post test for multiple comparisons, as appropriate. Values of P<0.05 were considered as statistically significant.

## Results

### Lipid Profile Following Ischemia

**Figure 1:**
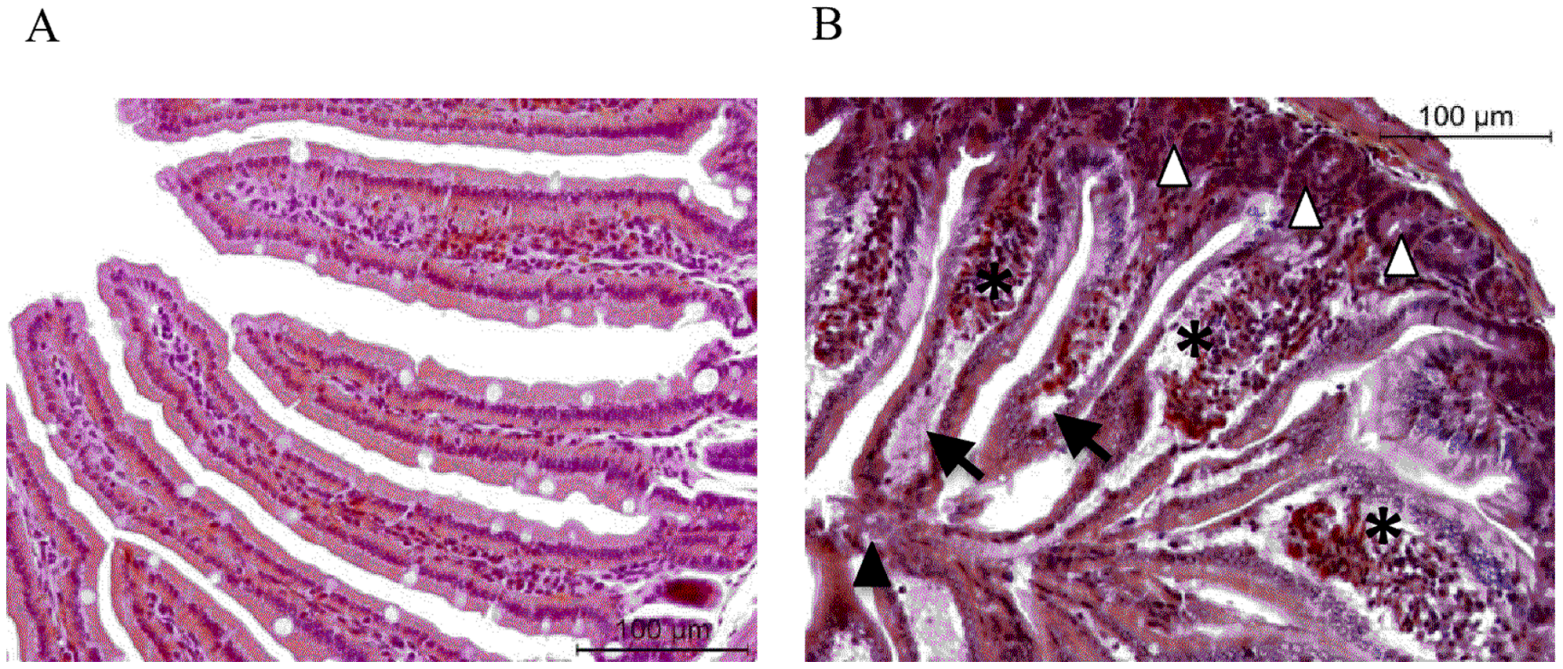
Effects of ischemia on mouse jejunal tissue. C57Bl6 mice were subjected to intestinal ischemia and sacrificed 50 minutes after the vessel occlusion. **A–B**, Histological examination of haematoxylin and eosin-stained sections of jejunal tissue. **A**, In the control mice (naïve or sham-operated) the mucosa had normal morphology. **B**, After 50 minutes of ischemia, considerable detachment of the epithelium from the villi (black arrows), dilated capillaries filled with erythrocytes (stars), and necrotic epithelia in the lumen (black arrowheads) were observed. Depletion of goblet cells was also evident. Gland architecture was intact (white arrowheads). Photographs are representative of 6 to 8 mice per group.

Ischemia condition (50 minutes) damaged the intestinal mucosa as observed by histology of the small bowel in Figure 1 (A and B). Considerable detachment of the epithelium from the villi (Guggenheim’s spaces) (black arrows Fig 1B), dilated capillaries filled with erythrocytes (stars Fig. 1B), and necrotic epithelia were observed (black arrowheads Fig. 1B). Depletion of goblet cells was also evident.However, gland architecture was intact (white arrowheads Fig. 1B). As previously described [10], intestinal MPO activity showed that granulocyte recruitment had not yet occurred (not shown). The experimental condition of the ischemia performed allowed 100% animal survival.

**Figure 2:**
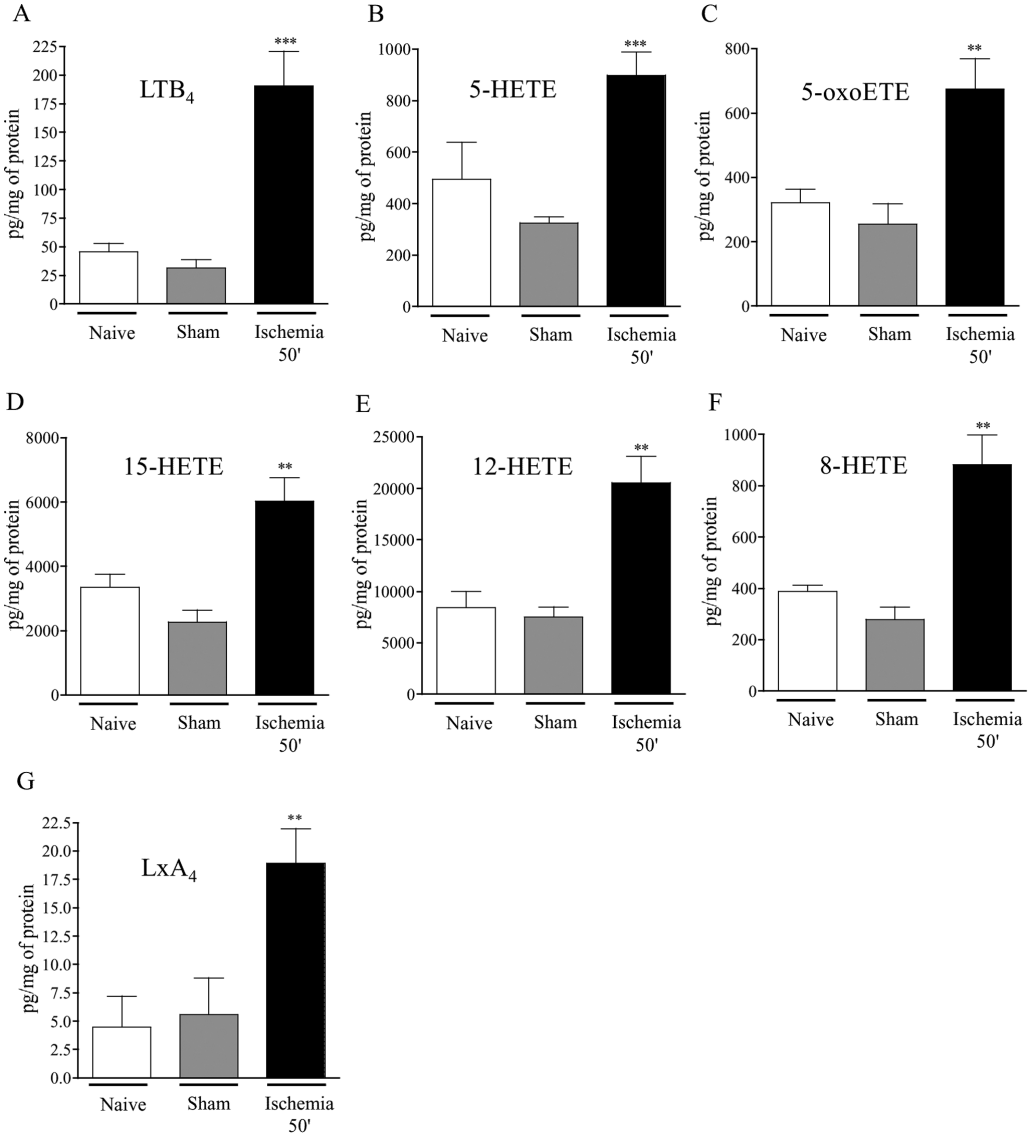
Effect of ischemia on intestinal LOX-derived eicosanoids production. Synthesis of eicosanoids from arachidonic acid (AA) was measured by liquid chromatography-tandem mass spectrometry in control mice (naïve and sham operated mice) and following 50 minutes ischemia. Data represent means ± SEM of 6 to 8 mice per group. *p<0.05, **p<0.01, and ***p<0.001 *versus* the corresponding sham operated group.

The effect of ischemia on intestinal eicosanoids synthesis derived from arachidonic acid (AA) or its precursor the dihomo-γ-linolenic acid (DGLA) is shown in table 1 and Figure 2.

**Table 1:** Effect of ischemia on intestinal COX-derived eicosanoids production.

	6kPGF1α	TXB_2_	PGE_2_	8isoPGA_2_	15d-PGJ_2_	PGA_1_
	AA					DGLA
Naive	8641±1337	2430±497.5	4495±664.4	95.35±23.38	2,334±1.17	7.656±2.90
Sham	7957±776.9	2180±179.5	3311±291.1	95.11±11.78	4.587±1.05	7.348±1.28
Ischemia	11496±1345*	2544±398	5310±653.8*	131.7±25.11	2.416±0.80	13.11±1.17*

Synthesis of eicosanoids from arachidonic acid (AA) or its precursor the dihomo-γ-linolenic acid (DGLA) was measured by liquid chromatography-tandem mass spectrometry in control animals (naïve and sham operated animals) and following 50 minutes of ischemia. Data are expressed in pg/mg protein and represent means ± SEM of 6 to 8 animals per group. *P<0.05 *versus* sham-operated group.

Jejunal PUFA metabolites derived from COX activation such as 6kPGF_1α_, PGE_2_ and PGA_1_ were significantly increased following ischemia compared to sham group. Conversely TXB_2_, 15d-PGJ_2_ derived from COX oxidation and 8-isoPGA_2_ derived from free radical oxidation were not significantly increased after 50-min occlusion of mesenteric artery. AA metabolites produced by lipoxygenase pathways were significantly increased (Figure 2 A–G). The synthesis of 8-HETE metabolized by 8-ALOX (15-LOX-2), 12-HETE metabolized by 12-LOX (R and S-type) and 15-HETE metabolized by 15-LOX were significantly increased (about 3-fold). The 5-LOX pathway constitutes the initial enzymatic step to generate 5-hydroperoxy-eicosatetraenoic acid (5HpETE). It can be reduced to 5-HETE, which can be further oxidized in 5-oxo-ETE, or rearranged in LTA_4_, which is the precursor of the potent chemoattractant LTB_4_ and of the anti-inflammatory LxA_4_. Ischemia significantly increased 5-HETE and 5-oxo-ETE production compared sham group. One of the most striking increases in PUFA metabolites upon ischemia in intestinal mucosa was the production of LTB_4_ (6-fold increase compared to sham). Rapid LxA_4_ generation was also detected at the end of the ischemic period.

**Figure 3:**
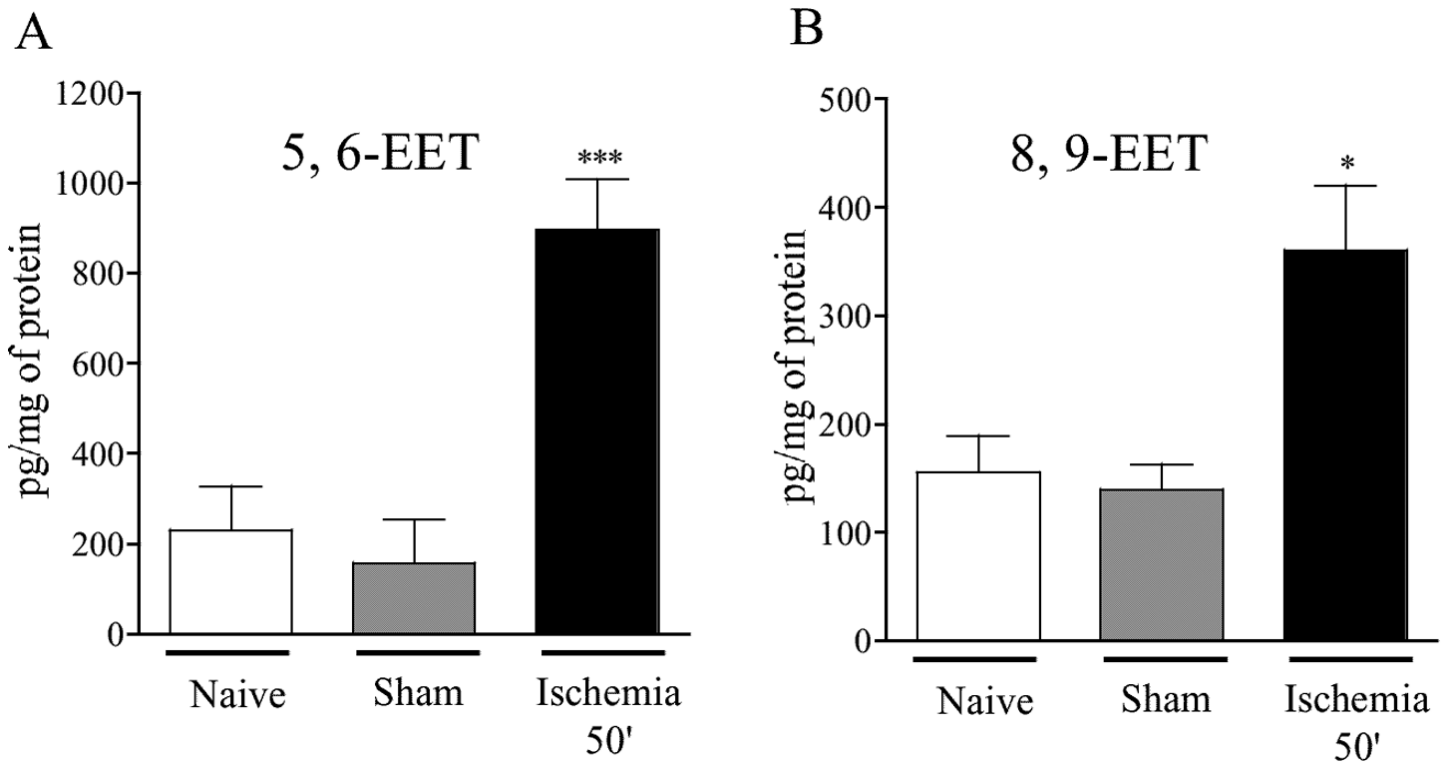
Effect of ischemia on intestinal CYP-derived eicosanoids production. Synthesis of eicosanoids from arachidonic acid (AA) was measured by liquid chromatography-tandem mass spectrometry in control mice (naïve and sham operated mice) and following 50 minutes of ischemia. Data represent means ± SEM of 6 to 8 mice per group. *p<0.05, and ***p<0.001 *versus* the corresponding sham operated group.

Epoxyeicosatrienoic acids (EETs) are major products of AA metabolism through the activation of cytochrome P450 (CYP) epoxygenase. Ischemia significantly increased 5, 6-EET and 8, 9-EET levels (5.7 and 2.5-fold respectively), compared to sham group. 11, 12-EET and 14, 15-EET were not detected (Figure 3).

**Figure 4:**
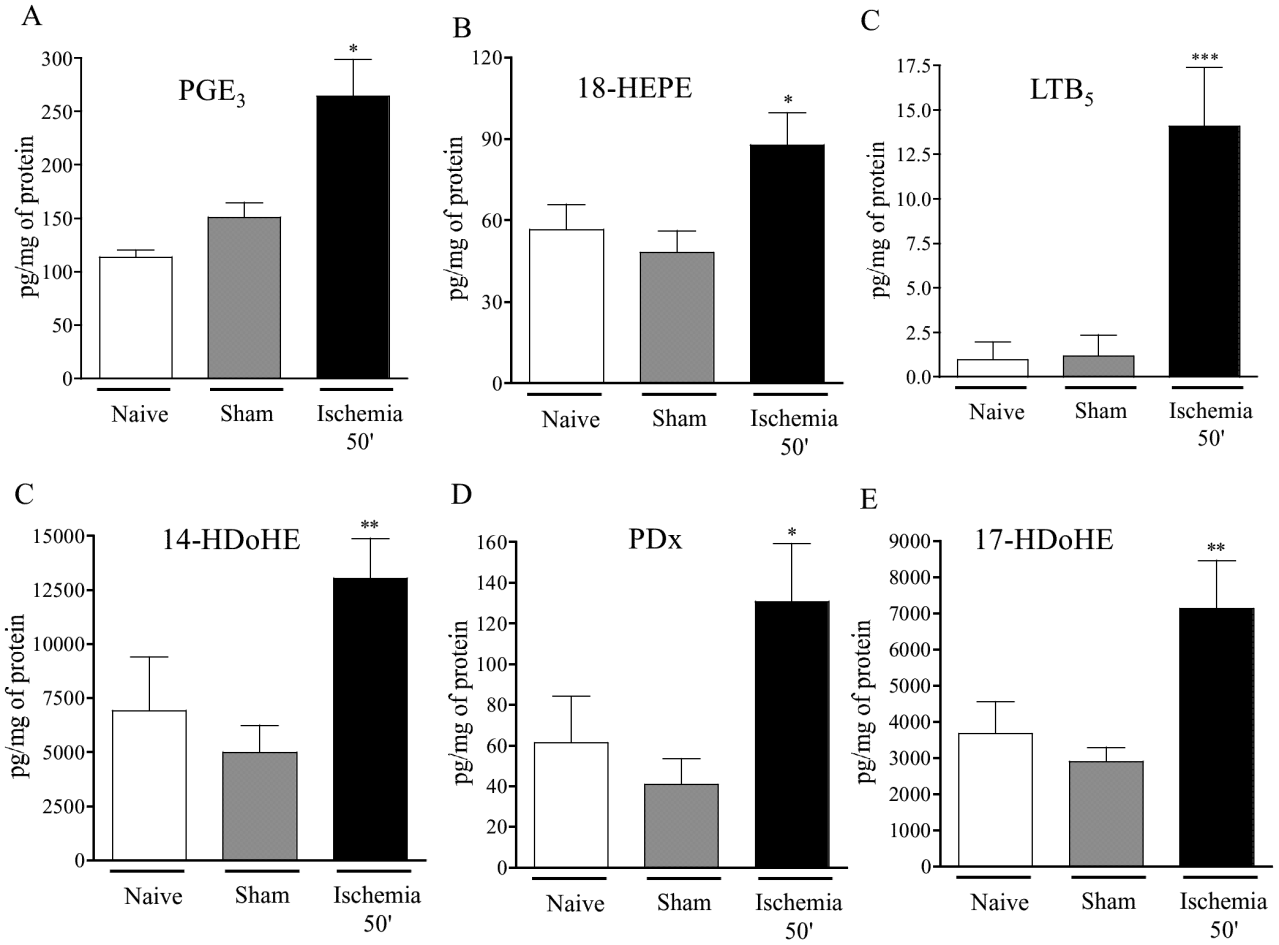
Effect of ischemia on intestinal docosanoid metabolites production. Synthesis of docosanoids from eicosapentaenoic acid (EPA) and docosahexaenoic acid (DHA) was measured by liquid chromatography-tandem mass spectrometry in control mice (naïve and sham operated mice) and following 50 minutes of ischemia. Data represent means ± SEM of 6 to 8 mice per group. *p<0.05, and **p<0.01 *versus* the corresponding sham operated group.

PUFAs n-3 such as EPA and DHA, even if they are poor substrates compared to AA, are susceptible to COX and LOX enzymatic metabolism. In excess of AA presence in the tissues, these *n*-3 fatty acids are very susceptible to free radical oxidation [12] PGE_3_ and 18-HEPE (the precursor of Resolvin E) derived from EPA by COX metabolism and free radical oxidation respectively were significantly increased after intestinal ischemia, compared to sham group (Figure 4). 14-HDoHE, a DHA oxidation product, 17-HDoHE, the precursor of both RvD and PD, and PD itself were significantly increased (2.7, 3 and 2.5-fold respectively), compared to sham. RvD1 was not detected (Figure 4).

All together, these data suggest that ischemia condition alone produces PGE_2_ and 6kPGF_1α_ but also massively activates LOX and CYP pathways, fed by omega-6 lipids. Ischemia alone also significantly increased enzymatic and non-enzymatic omega n-3 metabolism.

### Lipid Profile Following Reperfusion

**Figure 5:**
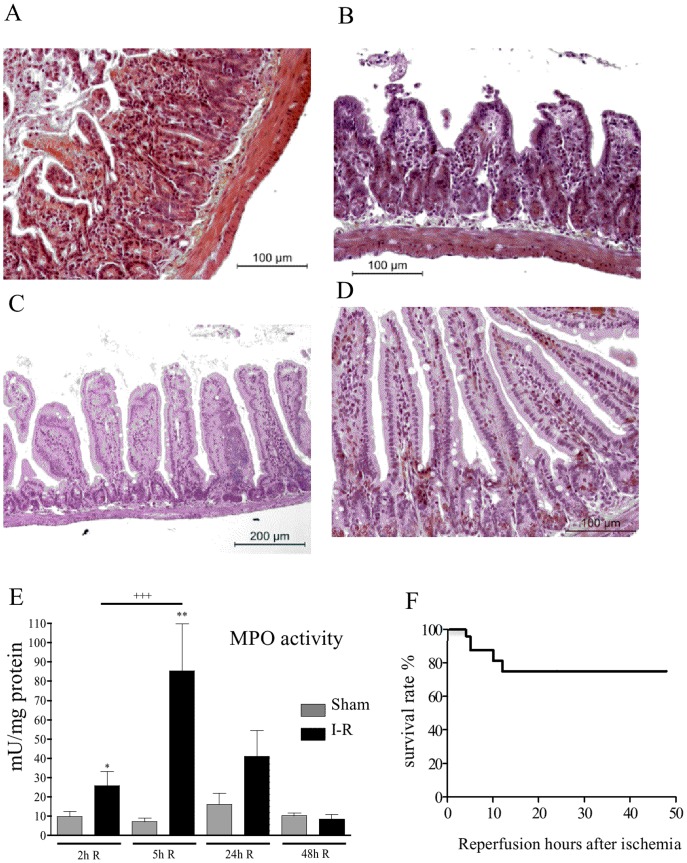
Effects of ischemia followed by reperfusion from 2 to 48 hours. A–D Histological examination of haematoxylin and eosin-stained sections of jejunal tissue. **A**, At 2 h reperfusion after ischemia, villi were severely damaged and the gland architecture was lost. **B**, At 5 h of reperfusion after ischemia, mucosal damage was still present even if a considerable re-epithelialization has yet occurred. However, villi remained flattened and epithelial cells were cuboidal in contrast with their usual columnar appearance. **C**, 24 hours after reperfusion, villi were reformed although shorter than villi from sham tissues. **D**, 48 hours after reperfusion, the mucosa appeared completely normal. **E**, As shown by intestinal MPO activity measurement, index of granulocyte recruitment, accumulation of inflammatory cells in the mucosa occurred as early as the first 2 h of reperfusion and was markedly increased at 5 h. **F**, Survival rate, the conditions of ischemia and reperfusion allowed 75% survival 48 hours after the surgery. Data represent means ± SEM of 6 to 8 mice per group. *p<0.05, and **p<0.01 *versus* the corresponding sham operated group; +++p<0.001 *versus* the indicated I–R group.

Histological injury produced by reperfusion was more severe than the damage induced by ischemia alone. At 2 h reperfusion after ischemia, villi were severely damaged and the gland architecture was lost. Accumulation of red blood cells (thrombi) was seen in the villus core (where villi remained). Fragments of mucosa and red cells could be found in the lumen. At 5 h of reperfusion after ischemia, mucosal damage was still present even if a considerable re-epithelialization has yet occurred. However, villi remained flattened and epithelial cells were cuboidal in contrast with their usual columnar appearance. One day after reperfusion, villi were reformed although shorter than villi from sham tissues. Two days after reperfusion, the mucosa appeared completely normal (Figure 5 A–D). Sham operations did not affect the mucosa architecture, which appearance was similar to tissues in Fig. 1A. As observed in figure 5E by MPO activity measurement, accumulation of inflammatory cells in the mucosa occurred as early as the first 2 h of reperfusion and was markedly increased at 5 h. At one-day after reperfusion, MPO activity was not significantly different from sham-operated mice. After 48 h reperfusion, MPO activity was similar to sham animals. Occlusion of superior mesenteric arterial blood flow for 50-min followed by reperfusion affected the survival of animals during the reperfusion period. The conditions of ischemia and reperfusion allowed 75% survival 48 hours after the surgery. Mortality was observed between 3 and 12 hours of reperfusion (figure 5F).

**Figure 6:**
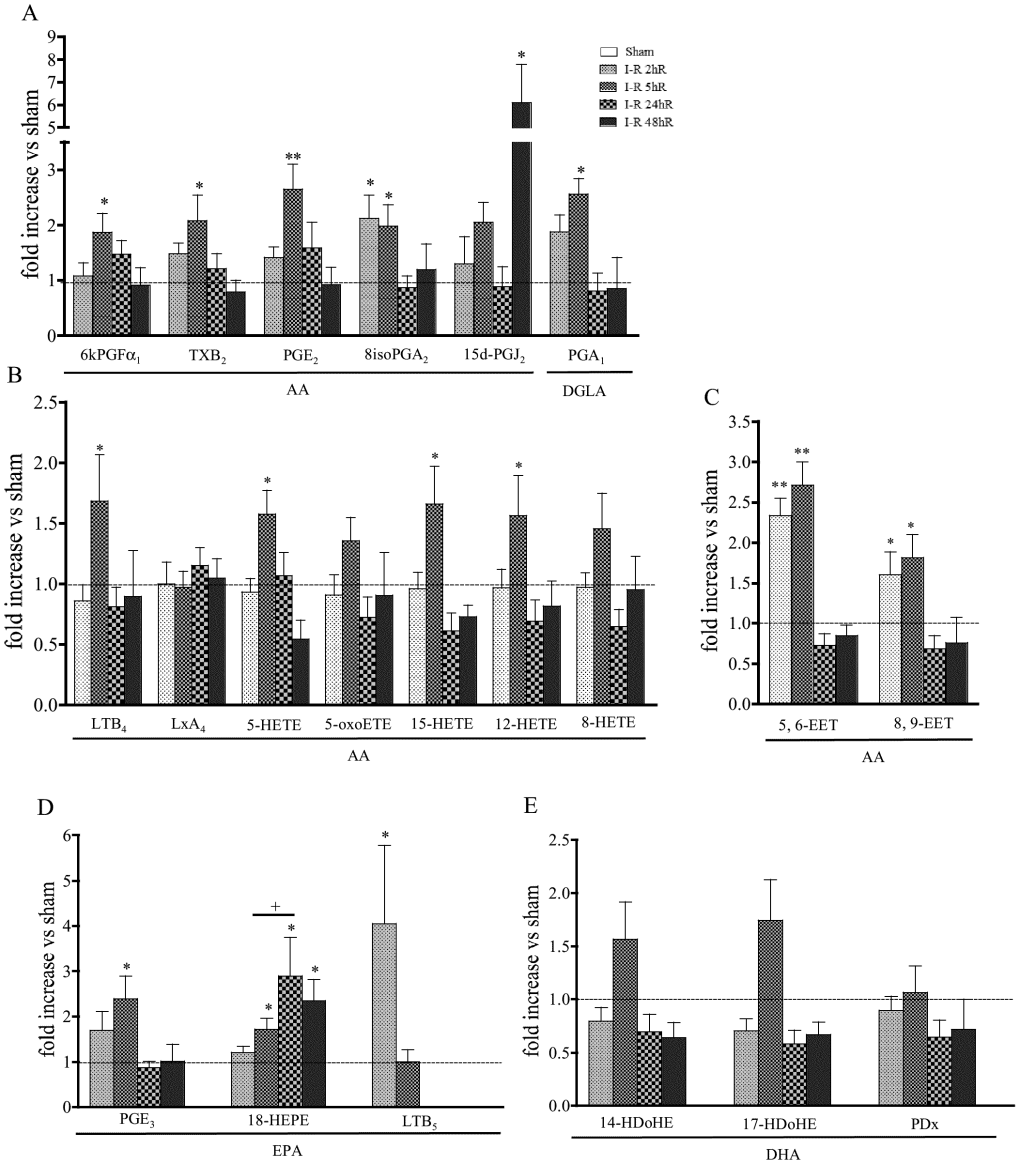
Effect of ischemia followed by reperfusion from 2 to 48 hours on intestinal eicosanoids/docosanoid production. **A**–**D** Synthesis of eicosanoids derived from COX-(**A**) LOX-(**B**) CYP-(**C**) arachidonic acid (AA) or its precursor the dihomo-γ-linolenic acid (DGLA) metabolism. **D,** Synthesis of docosanoid derived from eicosapentaenoic acid (EPA) and docosahexaenoic acid (DHA) metabolism. Data are expressed as fold increase *versus* corresponding sham operated group and represent means ± SEM of 6 to 8 mice per group.

Generation of PUFA n-6 metabolites in the intestine following reperfusion is shown in Figure 5. The well characterized metabolites derived from COX metabolism of AA, such as TXB_2_, 6kPGF_1α_, PGE_2_, and 15d-PGDJ_2_, were progressively increased by reperfusion up to a significant level compared to sham at 5 h after reperfusion. The 8-isoPGA_2_ and PGA_1_ were significantly increased (2-fold) after 2 and 5 h reperfusion. At 24 h after reperfusion, lipid mediator levels were not significantly different from sham and at 48-h after reperfusion, they fully returned to basal levels, except for 15d-PGDJ_2_ which was 7-fold increased after 48 h reperfusion (Figure 6A).

ALOX metabolites were not increased after 2 h of reperfusion compared to the corresponding sham group. A significant increase was shown at 5 h after reperfusion for LTB_4_, 5-HETE, 15-HETE and 12-HETE, although this increase seemed to be lower than in ischemia conditions. Twenty-four hours after reperfusion, all these metabolites returned to basal levels (Figure 6B). LxA_4_ was not increased by reperfusion compared to the corresponding sham group.

In contrast to ALOX metabolites, the CYP metabolites 5, 6 and 8, 9-EET followed the same pattern that was observed upon ischemia: they were significantly increased at 2 h and 5 h after reperfusion. At 24 and 48 h after reperfusion, EETs levels were not different from sham-operated mice. 11, 12 EET and 14, 15 EET were not detected, similar to the ischemia pattern (Figure 6C).

Concerning EPA metabolites (figure 6D), PGE_3_ was progressively increased starting from 2 hours after reperfusion and up to 5-h, by then it was increased by 2.2 fold. The precursor of Resolvin-D, 18-HEPE, was significantly increased in a time-dependent manner during the reperfusion. A peak was reached at 24 h reperfusion during the resolution phase of inflammation and was still increased compared to the corresponding sham at 48 h reperfusion. DHA metabolite 14-HDoHe and 17-HDoHE were increased at 5 h reperfusion only, but this increase was not significant. PD levels were unchanged at all time points of reperfusion, compared to sham group (figure 6E).

### TRPV4 Antagonist Aggravates the Post-ischemic Intestinal Inflammation

Based on the fact that ischemia and the first hours of reperfusion are associated with a strong release of 5, 6-EET, which has been proven to be an endogenous TRPV4 agonist [13], we investigated the effects of TRPV4 blockade on intestinal inflammation induced by 50′ minutes of ischemia, followed by 5 hours of reperfusion.

**Figure 7:**
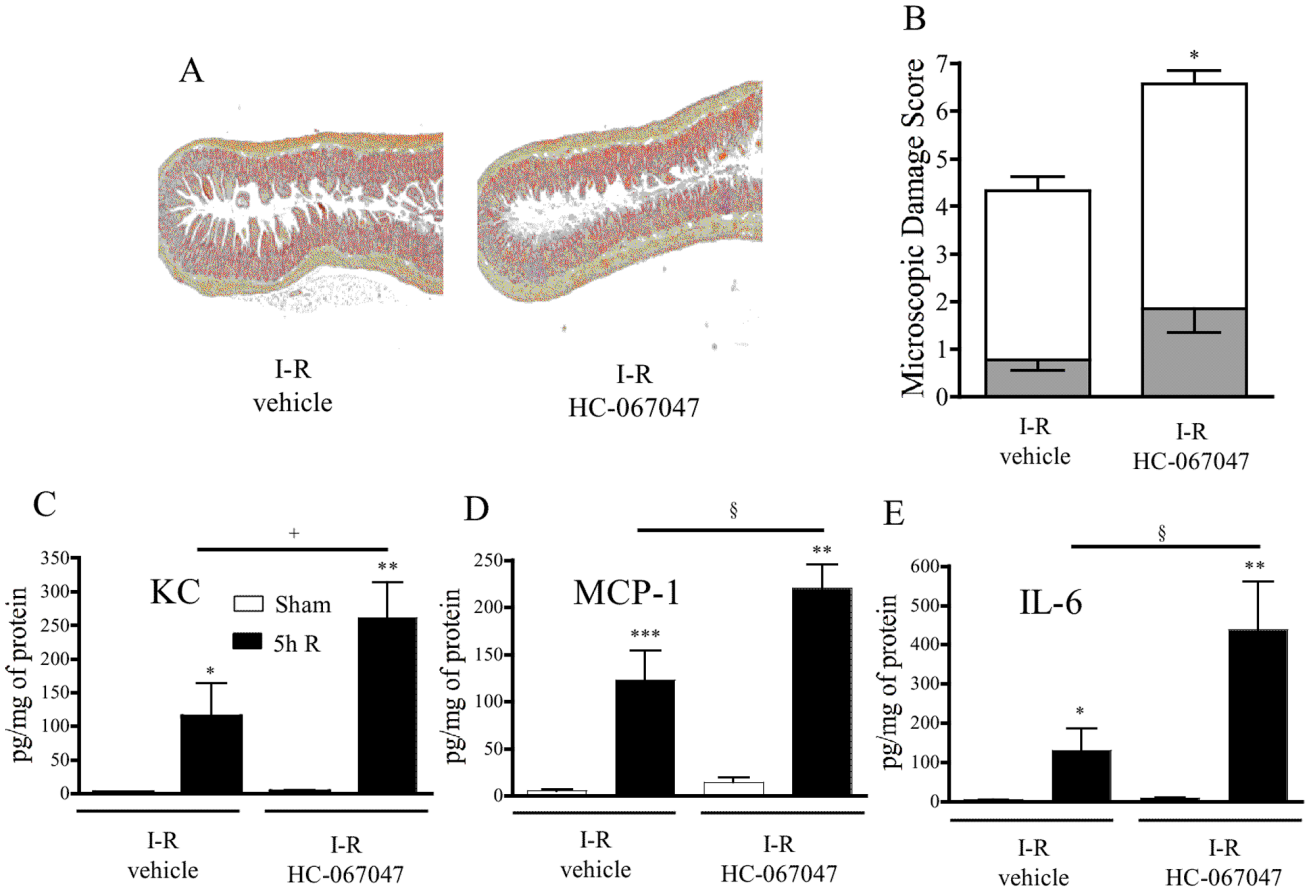
*In vivo* effects of systemic treatment with the transient receptor potential vanilloid-4 antagonist HC-067047 (50 mg/kg i.p.) or its vehicle. A, histological damage; B, microscopic damage (white) and MPO activity (grey); C–E chemokine (KC, MCP-1 and IL-6) tissue protein expression. Data in B, C, D and E represent means ± SEM of 6 to 8 mice per group. *p<0.05, **p<0.01, ***p<0.001 *versus* the corresponding sham operated group +p<0.05 *versus* the indicated I–R group.

Systemic treatment with the antagonist HC-067047 (50 mg/kg i.p.) significantly aggravates microscopic damage score as shown in figure 7A and B. The jejunal mucosa was more seriously ulcerated: an increased number of villi were flattened, more inflammatory cells were infiltrated, wall oedema and more red blood cells in villus core were evident compared to I-R vehicle group. The increased inflammatory response following I-R was supported by increased levels of KC, MCP-1 and IL-6 in jejunal tissues, compared to vehicle-treated mice (Fig. 7C–E). These data demonstrate that endogenous TRPV4 activation following I-R could modulate the post-ischemic inflammatory response in mice.

## Discussion

In the present study, using liquid chromatography-tandem mass spectrometry (LC-MS/MS), we provide an *in vivo* picture of time-dependent n-3 and n-6 PUFAs-derived metabolites production during intestinal ischemia-reperfusion injury. The present study provides important knowledge on the types of n-6 (AA or his precursor DGLA) and n-3 (EPA or DHA) PUFA metabolites that could orchestrate the ischemic and post-ischemic intestinal inflammation from its induction to its self-resolution. We identify here the metabolites and preferred metabolic pathways engaged in ischemia and reperfusion processes. Ultimately, these results could define new potential targets associated with ischemia-reperfusion injury and could help better choices of treatment.

Lipid molecules coming from PUFAs oxidation have emerged as very early initiator of sequential inflammatory cascade. They are released before that cytokines, chemokines or peptides further amplify the inflammation. This common principle is related to the ultra rapid production of eicosanoids or docosanoid, whereas the expression of other protein mediators is usually slower and controlled at transcriptional and translational levels requiring more time [14]. Indeed, we observed in our study a number of striking changes in PUFA metabolites and that was only upon 50 min of ischemia and few hours of reperfusion.

**Figure 8:**
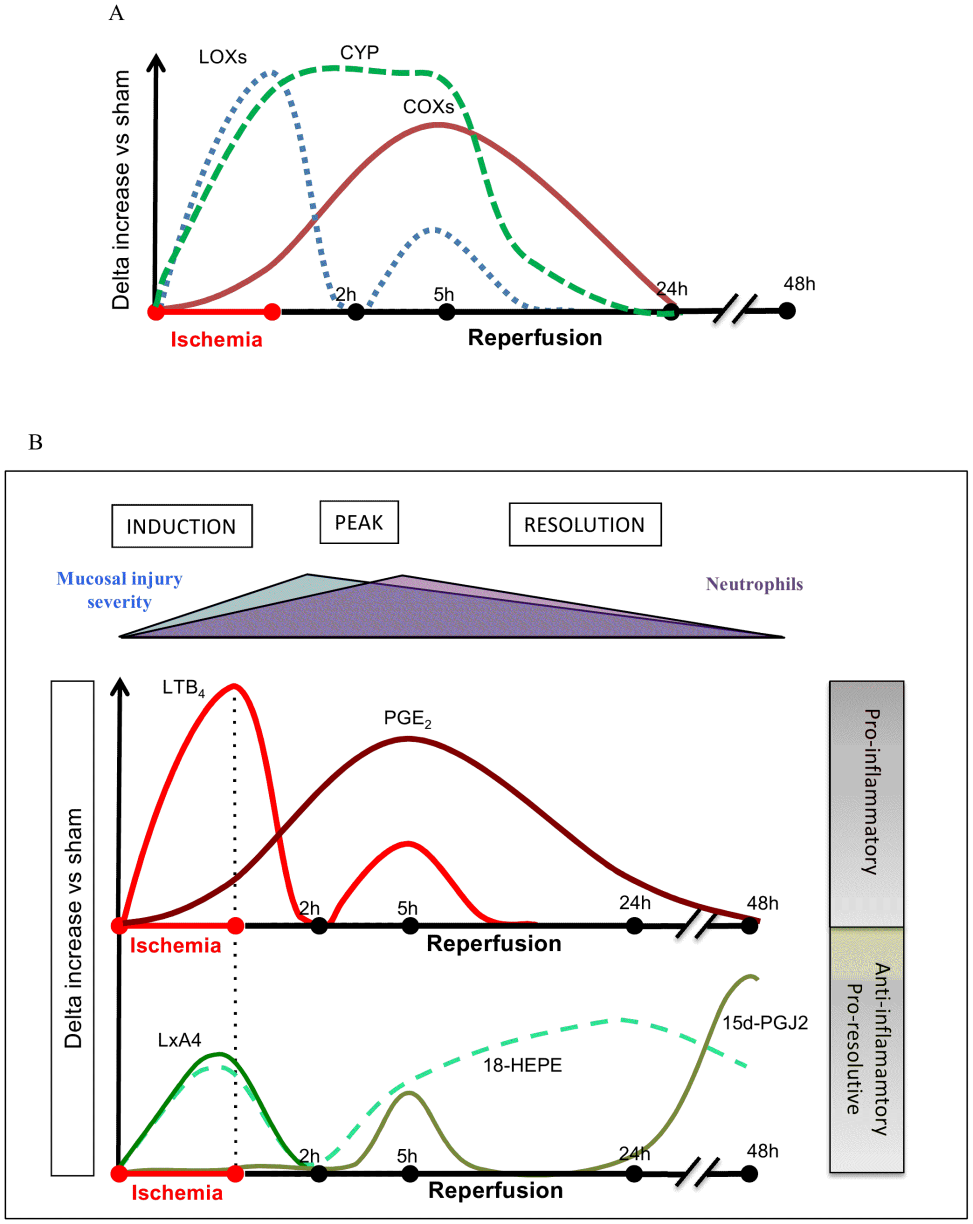
Temporal schemes of PUFA-producing enzymes and metabolites upon ischemia-reperfusion. **A**, Kinetic scheme of COX, LOX and CYP activation based on PUFA metabolites enzymatic biosynthesis. Early ischemia induces LOX metabolite biosynthesis, while COX activation seems to play a major role during the first hours after reperfusion (2 and 5 hours). CYP-derived metabolite synthesis starts immediately during ischemia and up to 5 hours reperfusion. **B,** Scheme of temporal PUFA metabolites production during intestinal ischemia reperfusion injury. Ischemic episodes (induction of the inflammatory response) lead to a concomitant early production of both the neutrophil chemo-attractant LTB_4_ and the vascular-protective LxA_4_. Immediate biosynthesis of LxA_4_ could assure an appropriate counterbalance role against ischemic damage. From 2 hours and up to 5-h reperfusion, PGE_2_ (such as other COX-derived metabolites) production was strongly increased fitting with the concomitant peaks of mucosal damage (2 hours) and granulocyte recruitment (5 hours). LTB_4_ (such as other LOX-derived metabolites) again significantly increased after 5 h of reperfusion, suggesting that at this time-point, additional cell source (potentially granulocytes) is responsible for the biosynthesis of LOX metabolites. At 24-h after reperfusion, all PUFA metabolites were decreased, to reach basal levels after 48 h of reperfusion, except for mediators known to take part into the resolution of inflammation: the RvE precursor 18-HEPE and the PPARγ agonist, 15d-PGJ_2_.

Unregulated calcium influx, oxidative stress and cell swelling associated with ischemia/hypoxia activate PLA_2_, which is particularly highly concentrated in the gut [15], [16]. Moore and his collaborators have already pointed out that PLA_2_ is extremely active following mesenteric artery occlusion and plays a pivotal role in the pathogenesis of intestinal ischemia-reperfusion injury [17], [18], [19]. Our results are also in favour of the hypothesis implicating PLA_2_ in intestinal ischemia-reperfusion. Indeed, several metabolites that could derive from PLA_2_ activity are released in hypoxic and re-oxygenated intestinal tissues. Further, we describe here the major metabolic pathways downstream from PLA_2_ activation that are activated during intestinal ischemia and reperfusion. First of all, our data suggest that a general strong increase in arachidonate oxidation by LOXs (as shown by LTB_4_ and HETEs levels) occurs in the intestine following ischemia alone. This LOX pathway was favoured compared to COX metabolism (see Figure 8). These results may suggest a pathogenic role for these eicosanoids in causing hypoxia-dependent injury such as impairment of endothelial cell barrier function, and immediate increase in vascular permeability. Indeed, the presence of early oedema was observed in histological pictures (Figure 1) after ischemia. LOXs are a family of enzymes that insert molecular oxygen into polyunsaturated fatty acid such as AA. In mice, LOXs can be classified according to diverse enzymatic activity in 5-LOX, 12/15-LOX (15-LOX type 1 for human), and 8-LOX (15-LOX type 2 for human) [20]. A key role of 5-LOX has already been shown in the pathogenesis of intestinal IRI [21]. Roles for 12- and 15-LOX pathways would have to be further investigated during ischemia, since we show here that metabolites from those pathways are produced in quantity. Our results demonstrate that metabolite products from LOXs pathways are mostly synthesized during hypoxic period more than during the reperfusion. If LOX inhibitors would have to be used as therapeutic options to limit inflammatory damage, our results provide evidences that those therapies would have to be applied early before or during the ischemic period rather than over the perfusion period. Finally, because our results show that LOX metabolites are produced mostly during ischemia, while infiltration of inflammatory cells to the tissues has not occurred yet, as demonstrated by low MPO activity (not shown), we can surmise that resident cells within the tissues are responsible for the synthesis of leukotrienes and other LOX-derived metabolites. This release of LOX-derived metabolites most likely set the stage for later leukocyte recruitment [22], as observed 2 h and 5 h after reperfusion (Figure 4). Although several *in vitro* studies have suggested that 5, 12 and 15-HETE may be involved in pro-inflammatory actions such as chemotaxis, migration of inflammatory cells, leukocyte vascular adherence and increased vascular permeability [23], [24], our results suggest for the first time a potential role for them *in vivo*, in ischemia reperfusion injury. Surprisingly, at the beginning of the reperfusion (2 h) LOX metabolite production was not increased compared to sham. After 5 h of reperfusion, LTB_4_, 5, 15 and 12-HETE were increased *de novo*. This increase in LOX-derived metabolites coincides with strong granulocyte recruitment into the tissue. This fact could suggest that leukocytes recruited may represent an additional source for biosynthesis of these metabolites.

Considering the COX-derived products dosed, they were all increased, in a time-dependent manner at 2 and 5 h of reperfusion and returned to basal levels by 48-h after reperfusion. Only PGE_2_ and 6kPGF_1α_ (stable form of PGI_2_), coming from COXs AA oxydation, were significantly increased after 50-min of mesenteric ischemia. This can be explained by the fact that even if COX-1 is constitutively expressed in intestinal tissues, the inducible form COX-2 is turned on upon reperfusion time and potentially participates to the generation of all COX metabolites [25], [26], [27]. COX-2-derived metabolites could of course be pro-inflammatory signals, but some of them could also exert anti-inflammatory properties. Indeed, COX-2-derived 15d-PGJ_2_ is important for resolution of inflammation [28], COX-2-derived PGD_2_ is an early anti-inflammatory signal in experimental colitis [29], and COX-2-derived LXA_4_ is able to protect against mucosal injury [30]. These results clearly identify that COX pathway is mostly involved in the reperfusion process rather than in the ischemic process (Figure 7A). From those metabolites, several are known for their pro-inflammatory properties. Endothelial PGE_2_ and platelet derived TXA_2_ (dosed here in its stable form TXB_2_) act as classic pro-inflammatory products controlling local blood flow, while PGI_2_ exerts an opposite function to TX [31], [32], [33]. Endogenous production of 8isoPGA_2_ in a model of mesenteric vessel occlusion following 2 and 5 h reperfusion was shown here for the first time. 8isoPGA_2_ is a cyclopentenone isoprostane (IsoP), formed by free radical-mediated peroxidation of arachidonic acid, and is usually considered as a marker of oxidative stress. Although its bioactivity is poorly understood, it could exert a role in post-ischemic inflammation, as recently suggested [34].

PGA_1_ (coming from COX oxidation of AA precursor DGLA) increased from the ischemic period and at 2 and 5 h reperfusion. This mediator is known to exert anti-inflammatory properties by activating PPAR [35]. Interestingly, endogenous production of 15d-PGJ_2_, the dehydration end product of PGD_2_, was increased at 5 h and even more increased at 48 h, suggesting a role in the resolution of inflammation for this mediator and a role for return to tissue homeostasis. Here again, the PGD_2_ metabolite 15d-PGJ_2_ is a potent PPAR-*γ* agonist *in vitro*, and may serve here as an endogenous PPAR-*γ* ligand, which known to be protective in IR [36]. In addition to being a potent agonist of PPAR-*γ*, 15d-PGJ_2_ inhibits also the activation of the transcription factor nuclear factor (NF)-*κ*B, which might then participate to its anti-inflammatory action [37]. Taken together, these results show that COX-derived metabolites have dual actions: some might amplify the inflammatory reaction upon reperfusion, while others already prepare the resolution phase. These potential effects are consistent with the fact that COX metabolism is mostly activated upon reperfusion (Figure 7A).

Epoxyeicosatrienoic acids (EETs) are cytochrome P450 (CYP) epoxygenase metabolites of arachidonic acid. EETs exist as four region-isomers (5,6; 8,9; 11,12 and 14,15-EET) that are rapidly converted into less biologically active dihydroxyeicosatrienoic acids (DHETs) by soluble epoxide hydrolase (sEH) [1], [38]. In our model of intestinal ischemia, 5,6-EET and 8,9-EET were increased following ischemia only, but also after 2 and 5 h of reperfusion. 11,12 or 14,15-EETs were not detected. One cannot exclude that an ultra rapid metabolization by sEH of these compounds takes place in ischemic/reperfused tissues, which does not allow their dosage in the absence of sEH inhibition. The metabolism of EETs by sEH is also highly region-selective. Indeed, 14,15-EET is the preferred substrate, 11,12-EET and 8,9-EET are hydrolyzed at a significantly lower rate, and 5,6-EET is very poor substrate for this enzyme [39]. It is assumed that elevation of intracellular EETs by EETs administration or knocking out of soluble epoxide hydrolase (sEH) exerts cardioprotective effects against ischemia-reperfusion (IR) injury. This protective effect could involve modulation of ion channels like ATP-sensitive potassium channels (K_ATP_) [40]. Furthermore, EETs exert anti-inflammatory properties by acting as PPARγ agonists, as it has been shown in a laminar flow model *in vitro* [41]. All together, these studies suggest that intestinal EETs production after ischemia and ischemia/reperfusion may play a role by counterbalancing pro-inflammatory signals induced by ischemia. Our study shows that the activation of CYP pathways spans over both the ischemic and reperfusion periods (Figure 7A). Considering the previously described protective roles for CYP-derived metabolites in *in vitro* models of ischemia-reperfusion, pharmacological inhibition of those pathways could be highly detrimental. CYP metabolism involves a number of enzymes, and selective inhibitors of these enzymes are poorly available. Therefore, in order to investigate the potential roles of some of the CYP metabolites released *in vivo* in intestinal ischemia-reperfusion, one has to question the potential downstream effectors of CYP metabolites. Specifically, we were interested in a receptor activated by the CYP metabolite that is mostly increased in our model: 5,6-EET. This receptor is the transient receptor potential vanilloid-4 (TRPV4). TRPV4 is a widely expressed cation channel of the transient receptor potential (TRP) superfamily. It can be activated by physical stimuli such as cell swelling or innocuous warmth. 5,6-EET has been described as an endogenous agonist of TRPV4 [13]. The role of this receptor in I–R injury has never been investigated. In the intestine, TRPV4 is expressed on intestinal epithelial cells [42], endothelial cells, immune cells (lymphocytes, mast cells, macrophage) and neurons [43], [44]. Administering a TRPV4 antagonist before ischemia caused additional mucosal damage, and increased the release of cytokines (IL-6) and chemokines (KC and MCP-1) (Figure 6). These results demonstrate that endogenous activation of TRPV4 is protective against ischemia and reperfusion injuries. This is in accord with a potential protective role for 5, 6-EET, its endogenous agonist that is highly produced by intestinal tissues upon intestinal ischemia-reperfusion (Figure 5C). However, the protective role of TRPV4 in inflammatory injuries associated with ischemia and reperfusion could seem opposite to the pro-inflammatory effects that have been described for TRPV4 activation on neurons [45], [46], [47] or on enterocytes [42]. One explanation could be that TRPV4 antagonism on enteric neurons blocks afferent fiber activation, which in the case of vagal cholinergic pathway, has been shown to protect against splanchnic artery occlusion. The protective effect of vagal nerve stimulation is consistent with the inflammatory reflex described by Andersson & Tracey, where vagal neuron activation inhibits inflammatory cell activation [48].

It is now currently accepted that resolution of inflammation is a coordinated and active process that involves not only the effective removal of inflammatory stimuli but also the generation of specific mediators. Local acting pro-resolving n-6 (i.e. LxA_4_) and n-3-derived lipid mediators such as D and E series resolvins, prevent excessive inflammation, has antimicrobial and anti-apoptotic activity, thereby promoting the restoration of tissue integrity and function. In the context of ischemia reperfusion injury, confirming our results, it has recently been shown that a rapid generation of circulating endogenous LxA_4_, during ischemia modulates downstream vascular inflammatory responses evident during the reperfusion phase. Furthermore, the exogenous delivery of LxA_4_ attenuates IR-mediated inflammation in Fpr2/3+/+ (LxA_4_ receptor) but not Fpr2/3^−/−^ [49]. Among the fatty acid metabolites, the n-3 PUFAs possess the capacity to control the resolution of inflammation by inducing the synthesis of local acting mediators with potent anti-inflammatory and immunomodulatory activities [50]. Therapeutically administered DHA, or direct infusion of Rv and PD decreased post ischemic inflammatory damage in a rat model of renal ischemia-reperfusion injury [51]. Resolvin E administration protects the heart from ischemic damage [52] Our data confirm the rapid generation of LxA_4_ and suggest that that EPA and DHA, similarly to AA, are immediately oxidized by enzymatic and non-enzymatic reactions upon the ischemic period. With the restoration of oxygen supply into the tissues, only PGE_3_ at 5 h and LTB_5_ at 2 h of reperfusion, which are known to exert less inflammatory effects than PGE_2_ and LTB_4_ respectively, as well as 18-HEPE were significantly increased in the gut. 18-HEPE is a stable precursor for Rv-E series, and was significantly increased over the entire period of reperfusion, even during the late events, where full tissue repair was achieved (48-h after reperfusion) (Figure 5D). Considering the implication of resolvins in the resolution of inflammation [53], these results fit with a role for 18-HEPE in mucosal repair. Collectively, these data suggest that DHA and EPA are quickly oxidized following intestinal ischemia, and they may serve as substrates for pro-resolving metabolites.

Based upon our analysis of lipid metabolites during IR, a kinetic scheme of enzyme activation can be proposed (Figure 8A). Early ischemia induced LOX metabolite biosynthesis that may take part to the pathogenesis of ischemic inflammatory damage. COX metabolites do not seem to be major metabolites implicated during ischemia. Concomitantly, EETs but also pro-resolving DHA and EPA metabolites are formed during ischemia probably playing a counterbalance role against ischemic damage. From 2-h and up to 5 hours after reperfusion, COX metabolites were strongly increased, fitting with the concomitant peaks of mucosal damage and granulocyte recruitment (Figure 8B). The release of LOX metabolites was not significantly increased compared to corresponding sham-operated mice after 2 h reperfusion. LOX metabolites were again significantly increased after 5 h of reperfusion, suggesting that at this time-point, an additional cell source (potentially granulocytes) is responsible for the biosynthesis of additional LOX metabolites. At 24-h after reperfusion, all PUFA metabolites were decreased, to reach basal levels after 48 h of reperfusion, except for mediators known to take part into the resolution of inflammation: 18-HEPE and 15d-PGJ_2_.

In conclusion, with the present study, we have characterized the specific profile of PUFA metabolites released upon ischemia and reperfusion, thereby providing a better comprehension of the kinetics of enzymatic pathways involved, and identifying metabolites that may play a role during those events. These results may help to consider the involvement of new receptors of PUFA metabolites and consequently could open the gate to the development of targeted therapies against ischemia and reperfusion-associated damage.
